# HENA, heterogeneous network-based data set for Alzheimer’s disease

**DOI:** 10.1038/s41597-019-0152-0

**Published:** 2019-08-14

**Authors:** Elena Sügis, Jerome Dauvillier, Anna Leontjeva, Priit Adler, Valerie Hindie, Thomas Moncion, Vincent Collura, Rachel Daudin, Yann Loe-Mie, Yann Herault, Jean-Charles Lambert, Henning Hermjakob, Tal Pupko, Jean-Christophe Rain, Ioannis Xenarios, Jaak Vilo, Michel Simonneau, Hedi Peterson

**Affiliations:** 1 Quretec Ltd., Ülikooli 6a, 51003 Tartu, Estonia; 2 Institute of Computer Science, University of Tartu, J. Liivi 2, 50409 Tartu, Estonia; 3 Swiss Institute of Bioinformatics, Vital-IT group, Unil Quartier Sorge, Genopode building, CH-1015 Lausanne, Switzerland; 4 CSIRO Data 61, 5/13 Garden St, Eveleigh, NSW 2015 Australia; 5 Hybrigenics SA, 3-5 Impasse Reille, 75014 Paris, France; 6 Institut national de la santé et de la recherche médicale, INSERM U894 2 ter rue d’Alésia, 75014 Paris, France; 7 Laboratoire Aimé Cotton, Centre National Recherche Scientifique, Université Paris-Sud, Ecole Normale Supérieure Paris-Saclay, Université Paris-Saclay, 91405 Orsay, France; 8 (Epi)genomics of Animal Development Unit, Institut Pasteur, CNRS UMR3738, Paris, 75015 France; 9 Centre Européen de Recherche en Biologie et Médecine, 1 rue Laurent Fries, 67404 Illkirch, France; 10 Institut Pasteur de Lille, UMR 744 1 rue du Pr. Calmette BP 245, 59019 Lille cedex, France; 11 European Molecular Biology Laboratory, European Bioinformatics Institute (EMBL-EBI), Wellcome Trust Genome Campus, CB10 1SD Hinxton, United Kingdom; 12 George S. Wise Faculty of Life Sciences, School of Molecular Cell Biology and Biotechnology, Tel Aviv University, P.O. Box 39040, 6997801 Tel Aviv, Israel; 13 Center for Integrative Genomics University of Lausanne, Genopode, 1015 Lausanne, Switzerland; 14 Genome Center Health 2030, Analytical Platform Department, Chemin des Mines 9, 1202 Genève, Switzerland; 15 DFR CHUV, Rue du Bugnon 21, 1011 Lausanne, Switzerland; 16 Agora Center, LICR/Department of Oncology, Rue du Bugnon 25A, 1005 Lausanne, Switzerland

**Keywords:** Regulatory networks, Alzheimer's disease, Scientific data, Data integration

## Abstract

Alzheimer’s disease and other types of dementia are the top cause for disabilities in later life and various types of experiments have been performed to understand the underlying mechanisms of the disease with the aim of coming up with potential drug targets. These experiments have been carried out by scientists working in different domains such as proteomics, molecular biology, clinical diagnostics and genomics. The results of such experiments are stored in the databases designed for collecting data of similar types. However, in order to get a systematic view of the disease from these independent but complementary data sets, it is necessary to combine them. In this study we describe a heterogeneous network-based data set for Alzheimer’s disease (HENA). Additionally, we demonstrate the application of state-of-the-art graph convolutional networks, i.e. deep learning methods for the analysis of such large heterogeneous biological data sets. We expect HENA to allow scientists to explore and analyze their own results in the broader context of Alzheimer’s disease research.

## Abstract (summary)

**Table:** 

Design Type(s)	data integration objective • disease analysis objective
Measurement Type(s)	Alzheimer’s disease
Technology Type(s)	digital curation
Factor Type(s)	
Sample Characteristic(s)	Homo sapiens • brain

Machine-accessible metadata file describing the reported data (ISA-Tab format)

## Background & Summary

Alzheimer’s disease (AD) is an age-related neurodegenerative disorder that progresses with age and eventually leads to death. Several approved drugs can be applied to reduce the symptoms of Alzheimer’s disease, however, no current treatments can modify the underlying disease processes^1,2^. A number of experiments have been performed to understand the regulatory mechanisms of the disease^3–6^. The results obtained from such experiments are collected in various databases that were created for depositing and providing further access to similar types of experimental biological data such as ArrayExpress, IntAct, hu.MAP, and ADNI^7–10^.

**Fig. 1:**
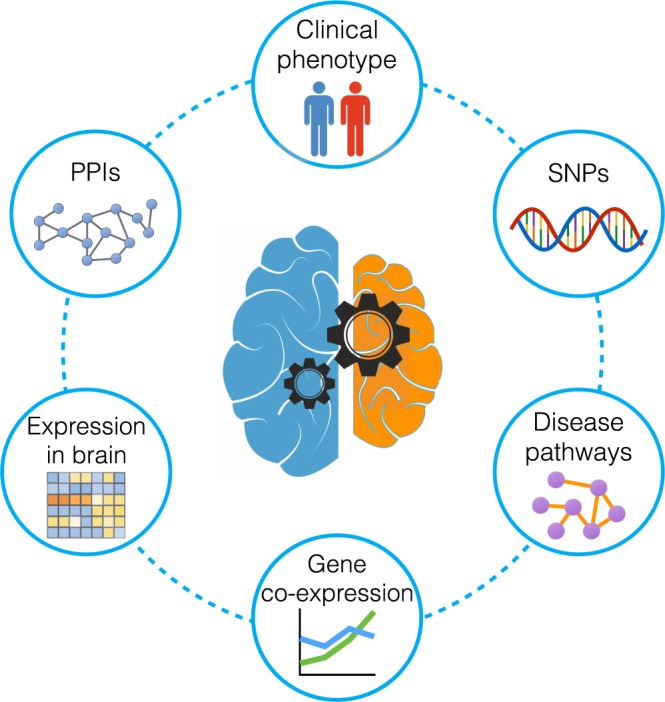
Bringing together heterogeneous data related to Alzheimer’s disease. Diagram represents the enrichment of the knowledge about Alzheimer’s disease by combing genomics, proteomics and clinical phenotype data.

The studies that utilize the results of such experiments address the causes of brain ageing by researching the mechanisms involved in this process. These studies are carried out to identify the molecular interactions through which the ageing phenotype develops in normal and disease conditions^11–13^. Analyses of various data types, like protein-protein interactions (PPI), gene expression, and medical imaging, have uncovered substantial information about disease mechanisms. The majority of such studies have focused on analysis of a particular experimental data type, resulting in the identification of several markers and biological interactions associated with Alzheimer’s disease^2,11,14–16^. However, since the disease-related data sets originate from many types of experiments, performed by scientists working in different domains such as proteomics, molecular biology, clinical diagnostics and genomics, these data sets are not collected in a single repository and format, making it difficult to obtain a systems view of the molecular elements and interactions that are involved in the disease (Fig. 1).

**Fig. 2:**
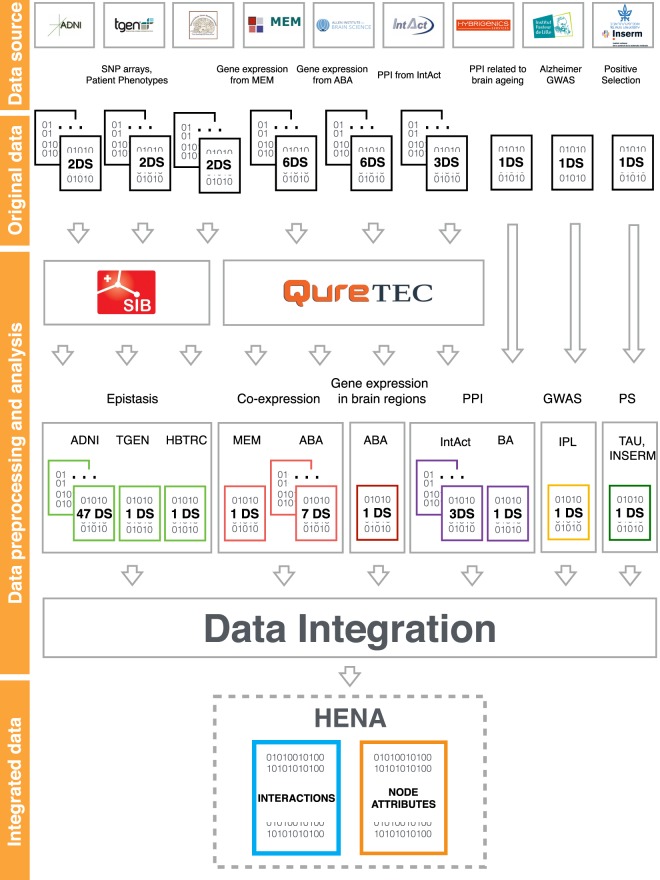
Project pipeline. HENA brings together 64 preprocessed computational and experimental data sets. Epistasis, co-expression and aggregated gene expression data sets were computed by SIB and Quretec using original data sets from ADNI, TGEN, HBTRC, MEM and ABA data sources. PPI from IntAct data source were preprocessed and added to the collection. Data sets generated and provided by the consortium members, i.e. PPI related to brain ageing, positive selection and Alzheimer’s GWAS were added directly during the integration step. Data sets are colored based on the data type. i.e. epistasis is colored light green, co-expression is red, aggregated gene expression is dark red, PPI is violet, GWAS is yellow and positive selection is dark green. The individual data sets are acknowledged by citing the DOI. The final integrated data set has separate DOI and data authors.

As an approach to overcoming such limitations in the case of research related to Alzheimer’s disease, we present here a HEterogeneous Network-based data set for Alzheimer’s disease. HENA results from an extensive data collection and is designed to allow prioritization of protein pairs using complementary information. It is accessible via the Network Data Exchange (NDEx) repository^17–19^ and via the figshare repository^20^. HENA was created to integrate Alzheimer’s disease-related data from well-known public data collections, as well as novel experimental and computational data sets generated by the members of the AgedBrainSYSBIO consortium^21^ (Fig. 2). We have generated computational data sets, i.e. data sets of epistatic and co-expression interactions, by utilizing data from Alzheimer’s disease-specific experimental data collections.

HENA combines 64 distinct computational and experimental data sets of six data types originating from nine data sources, as described in Online-only Table 1 and Fig. 2. These data types include protein-protein interactions, gene co-expression, epistasis, genome-wide association studies (GWAS), gene expression in different brain regions, and positive selection data. We combine selected Alzheimer’s disease data, taking into account its incompleteness, in an attempt to harmonize the existing large data collections.

One way of combining heterogeneous data sets of various types and formats is the transformation of these data sets into an intermediate form, such as a network^22–24^. This approach can be used to integrate many types of data as long as they contain a common unifying feature. We applied transformation-based integration by representing individual interaction data set in the form of an edge list and combine them into one network, where edges represent biological relations and nodes represent biological entities such as genes, SNPs and proteins. Additionally, we collected data sets containing Alzheimer’s disease-related information about SNPs, genes and proteins. These data sets were then combined to constitute a table of node attributes. Thus, HENA consists of a network of heterogeneous biological interactions and a table of node attributes.

Recent advances in biological network analysis methods, i.e. the application of graph convolutional networks (GCN), have demonstrated that network structure carries rich information helping to effectively uncover underlying disease mechanisms and pathways^23,25^. In the description of a use case, we demonstrate how combined heterogeneous data sets in the network format can help to develop a better understanding of Alzheimer’s disease.

## Methods

### Transformation-based integration overview

Bringing together individual data types undoubtedly enriches the systematic view on the Alzheimer’s disease, giving researchers the opportunity to understand molecular mechanisms from the genetic to protein levels. However, it also involves some limitations of biological data integration due to its heterogeneous nature^26^ such as different formats and standards, lack of common name space, incompleteness, versions of data collections, and size differences. Currently in the field of omics integration there is an open question about which molecular level should be used to provide a flexible and effective way to describe a biological system on different levels^22,26^. The answer to this question is highly dependent on the available omics data sets. Our approach is meant to allow the researchers to transit from one layer of information to the other by combining the individual layers of knowledge into one model. In our work we proposed an integration at the gene level, allowing researchers to go from SNPs to proteins to grasp more complete picture of the molecular interactions in Alzheimer’s disease.

**Fig. 3:**
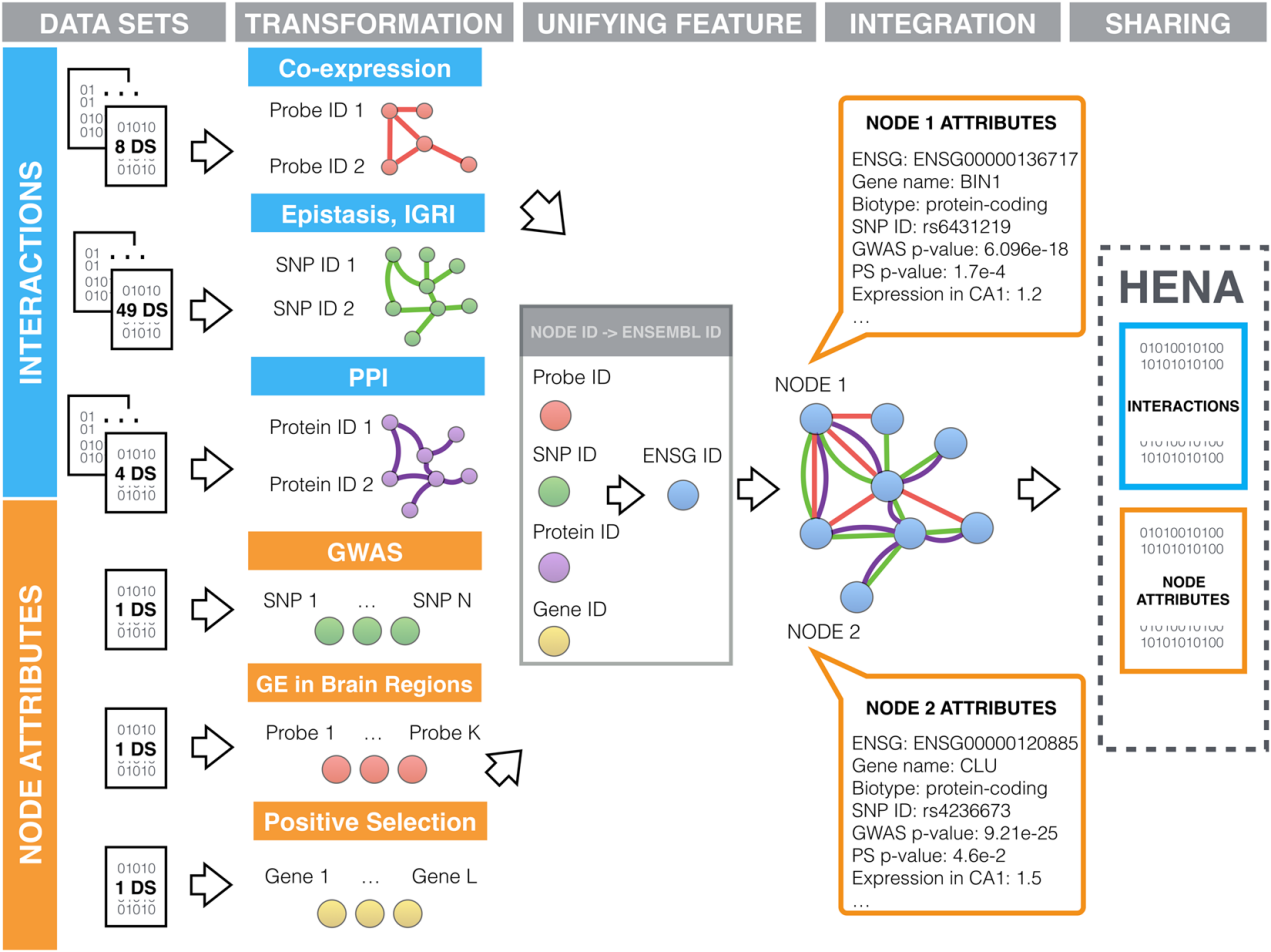
Data integration pipeline. Preprocessed data sets contain information about the interactions between genes, proteins, SNPs, and information characterizing them, e.g. node attributes. Interaction data sets contain PPI, co-expression and epistatic interactions including IGRI as its sub-type. Node attributes originate from GWAS, positive selection and gene expression in brain regions from ABA. Integration was performed using transformation-based approach. Data sets from the interaction group were converted into intermediate graphs, where nodes are genes, proteins, SNPs and the edges are the relations such as PPIs (violet), epistatic interactions (green) or co-expression interactions (red). All individual node identifiers were mapped at the gene level and converted into the ENSEMBL name space. Individual graphs were then combined into one heterogeneous graph with possible multiple edges between two nodes. Additionally, each node has been characterized by a set of attributes such as ENSG ID, corresponding gene name, gene biotype, SNP ID, GWAS p-value, positive selection p-value and aggregated expression in 231 brain regions.

We combine the heterogeneous experimental data from various sources into one heterogeneous network-based data set for Alzheimer’s disease. The HENA data set^18,19^ consists of two parts–a graph, describing biological interactions between genes, proteins, single nucleotide polymorphisms (SNPs), gene probes, and a set of node attributes providing additional information about the genes (Fig. 3). Gene interactions include protein-protein interactions, co-expression, and epistasis. Due to this variety of the interactions’ nature, the origin from various databases and experimental sources, there is a lack of unified node identifiers. To overcome this problem the identifiers of the proteins, SNPs, probes and gene names originating from various databases are mapped to one unique Ensembl name space^27^.

We represented each individual interaction data set in a form of the network (Fig. 3), where nodes stand for entities like proteins, genes, SNPs, etc., and where edges depict biological relations between the nodes, e.g. protein-protein interactions, co-expression, epistatic interactions. Multiple individual networks were then combined into one network using the unifying feature. As a unifying feature we used genes onto which SNPs, transcripts and proteins can all be mapped. We mapped identifiers from each individual data set such as SNP, protein and gene identifiers to a common name space. Due to the fact that genes or proteins coming from different databases might have a few alternative names, unique gene identifiers provided by Ensembl ver. 93 (ENSG ID) were used as a common name space. Each interaction A-B in the networks is described by the score, interaction type and the data set where the interaction can be found. A summary of edge attributes is presented in Table 1. Additional disease-related information about each node, e.g. GWAS association, positive selection, expression in the brain regions, is collected in the form of a table with the attributes described in Table 2. For each node a list of attributes is a vector of the length 237. In the sections below we describe how experimental and computational data sets were acquired, transformed and combined.

**Table 1:** List of edge attributes.

Edge attribute	Description
ENSG.A	ENSG IDs of the node A
ENSG.B	ENSG IDs of the node B
Score	Value, associated with the interaction. It represents the importance of an interaction, i.e. strength, significance.
Interaction type	Type of biological relation between node A and B, i.e co-expression, PPI, epistasis.
Data source ID in HENA	Short name of the data source of the interaction. In case multiple data sets originate from one data source, data source name is appended by individual data set.

**Table 2:** List of node attributes.

Node attribute	Description
ENSG	ENSG ID of the node
Gene name	Alternative human readable gene name.
Biotype	Gene biotype according to Ensembl classification i.e. protein coding, long non-coding, etc.
SNP ID	SNP identifier from GWAS data set.
GWAS p-value	P-value corresponding to SNPs from GWAS data set.
PS p-value	P-value of gene association with positive Darwinian selection.
Brain region ID #1	Aggregated gene expression of the node in brain region ID #1
…	…
Brain region ID #231	Aggregated gene expression of the node in brain region ID #231

### Co-expression analysis

Co-expression in Alzheimer’s disease and healthy individuals was computed based on the selected six Affymetrix GeneChip Human Genome U133 Plus 2.0 microarray gene expression data sets^28–33^ from the Multi Experiment Matrix (MEM) database^34^. Data sets were obtained in NetCDF format and stored as a “Gene expression microarray datasets in NETCDF format from MEM database” part of HENA data collection at figshare repository^20^. These data sets were chosen manually based on the sample annotations. Selected data sets contain samples from patients with neurodegenerative disorders including Alzheimer’s disease (AD) and healthy individuals (HI) (E-MEXP-2280^28^ (5 AD, 5 HI), E-GEOD-5281^29^ (88 AD, 73 HI), E-GEOD-4757^30^ (10 AD, 10 HI), E-GEOD-29652^31^ (18 AD), E-GEOD-28146^32^ (22 AD, 8 HI), E-GEOD-18309^33^ (3 AD, 3 HI)). For further analysis we have selected only the samples related to the patients with Alzheimer’s disease (AD) and healthy individuals (HI).

We calculated the Spearman correlation between all pairs of probes in each data set and sort-ranked them based on the correlation value. In each data set the pair with the strongest correlation value received the highest rank (rank 1). Ranks obtained for each pair of probe sets in each data set were then normalized and aggregated using the Robust Rank Aggregation (RRA) method implemented in Robust Rank Aggreg R package^35^. The final RRA scores were adjusted for multiple testing using FDR method. We kept only the pairs where the RRA score is smaller than 1*e*^−5^. Similar to the p-value, the RRA score varies from 0 to 1. The smaller the score, the stronger the association between the pair of genes is. We carried out the computation of RRA scores at the probe set level, and later mapped Affymetrix probeset names to Ensembl ver.93 gene name space (ENSG ID) using gProfileR R package ver. 0.6.2^36^. Multiple probe set names can correspond to the same unique ENSG ID. This one-to-many mapping problem has resulted in the presence of multiple correlated pairs consisting of genes with the same ENSG IDs but with different corresponding scores. Bearing in mind this issue, we have aggregated those pairs for the data set by selecting the maximum, i.e. the most conservative, value out of all scores in all duplicated pairs. Additionally, due to the non-symmetrical nature of the RRA scores for the pair of nodes A-B and B-A (where A and B are the correlated genes), we kept the most conservative value out of two scores. The resulting co-expression interactions were added to the HENA data set in a form of interaction between two nodes (ENSG.A–ENSG.B) and a score representing the RRA score (see Online-only Table 1 for details). The computations were executed in parallel at the HPC center of the University of Tartu using doParallel R package ver.1.0.14^37^.

### Co-expression in disease-related brain regions

Gene co-expression analysis is a widely used method for the identification of possible functionally related genes and potentially interacting proteins^38–40^.

In this study co-expression analysis was performed using six Agilent 8 × 60 K whole-brain gene expression microarrays^41–46^ downloaded from Allen Human Brain Atlas (ABA)^47^. Combined data sets were quantile-normalized and filtered to eliminate probes with low variance. Data quality control for batch effect was performed using principal component analysis^48^. It has been previously demonstrated that the hippocampal circuit consisting of cornu ammonis sectors (CA1-CA4), dentate gyrus (DG), subiculum, and septal nuclei (SptN) are affected by Alzheimer’s disease^1,49–54^.

We have extracted samples corresponding to these disease-associated regions from the combined data set using the tissue ontology provided along with the microarrays as a ref.^55^. The Spearman rank correlation coefficients and the corresponding p-values were computed for each probe pair in the data set using R package psych ver. 1.8.4^56^. All p-values were adjusted using FDR correction. To select the probes with the reliable co-expression, we have filtered the results. On the first filtering step only the co-expressed pairs with the p-value ≤ 0.01 were kept. Additionally, these resulting pairs were filtered based on the correlation coefficient, selecting values ≥1^st^ quartile of the positive correlation distribution and ≤3^rd^ quartile of the negative correlation distribution to be included in HENA. The computations were executed in parallel at the HPC center of the University of Tartu using doParallel R package ver. 1.0.14^37^.

The resulting interactions were added to the HENA data set in the form of an interaction between two nodes, ENSG.A and ENSG.B, and a score representing the Spearman correlation coefficient. Co-expressions in each disease-related region is reported separately (see Online-only Table 1 for details).

### Epistasis analysis

Epistatic effects were computed in three cohorts. The disease-associated traits that were used included the change in ventricular volume, derived from successive brain MRIs, scores from a panel of cognitive traits tests and Braak staging reflecting the disease severity^57^. The epistatic interactions were computed as the departure from addtitivity of the effects of pairs of SNPs on phenotype, as detailed below.

Epistatic interactions between pairs of SNPs associated with change in ventricular volume^20^ detected in ADNI consortium^10^ patients were computed based on genotyping and longitudinal brain imaging data that were downloaded from the ADNI database^58^ provided by the ADNI consortium. We have used data from the subset of 719 participants from the first phase of the ADNI project for whom both genome-wide genotype and MRI data were available.

The epistatic effects on quantitative traits of 400 Late-Onset Alzheimer’s Disease (LOAD) patients from ADNI cohort^20^ were detected analysing the relation between GWAS genotypes and the set of 23 cognitive traits regularly assessed in these patients. The list of all 23 cognitive traits is available in Table 3. Measurements of cognitive traits were available at time points which varied from one individual and trait to the other, in number, frequency, and relation with age or time of diagnosis. Bearing this in mind, two values were used for the assessment of each cognitive trait – CT_latest the last value assessed for each patient, and CT_slope the slope of the line fitted by linear regression to successive values assessed for each patient. Slope value serves as a quantification of the trend of a given trait, i.e. the rate at which it changes over time. Due to the fact that AD is a progressive disease, and the ADNI cohort is composed of individuals of various age and disease stage, such data derivation may be more significant than absolute instantaneous value, especially in individuals in the early stages of disease development. Altogether computation of the epistatis effects resulted in 23 × 2 data sets (see Online-only Table 1).

**Table 3:** List of cognitive traits in ADNI cohort.

Trait name	Abbreviation
Alzheimer’s Disease Assessment 11-items composite score	ADAS11
Alzheimer’s Disease Assessment 13-items composite score	ADAD13
Clinical Dementia Rating Scale	CDRSB
Everyday Cognition. Participant self report. Divided Attention	Ecog_PtDivatt
Everyday Cognition. Participant self report. Language	Ecog_PtLang
Everyday Cognition. Participant self report. Memory	Ecog_PtMem
Everyday Cognition. Participant self report. Organization	Ecog_PtOrgan
Everyday Cognition. Participant self report. Planning	Ecog_PtPlan
Everyday Cognition. Participant self report. Visuospatial	Ecog_PtVisspat
Everyday Cognition. Participant self report. Total	Ecog_PtTotal
Everyday Cognition. Study partner clinician report. Divided Attention	Ecog_SPDivatt
Everyday Cognition. Study partner clinician report. Language	Ecog_SPLang
Everyday Cognition. Study partner clinician report. Memory	Ecog_SPMem
Everyday Cognition. Study partner clinician report. Organization	Ecog_SPOrgan
Everyday Cognition. Study partner clinician report. Planning	Ecog_SPPlan
Everyday Cognition. Study partner clinician report. Visuospatial	Ecog_SPVisspat
Everyday Cognition. Study partner clinician report. Total	Ecog_SPTotal
Functional Activities Questionnaire	FAQ
Montreal Cognitive Assessment	MOCA
Mini-Mental State Examination	MMSE
Rey Auditory Verbal Learning Test. Immediate	RAVLT_Immediate

Epistatic interactions between pairs of SNPs associated with Braak staging in TGEN cohort^20^ were detected through a genome-wide analysis of epistatic effects in the AD case-control cohort available from the Translational Genomics Research Institute^59^. The TGen II cohort included 1599 Caucasian individuals (1,014 AD cases, 585 controls), collected by TGen, as has been described^60^. Data from 915 patients with available genotypes and Braak scores were used (613 AD cases, 302 controls).

Epistatic interactions between SNPs associated with Braak staging^20^ were computed in Harvard Brain Tissue Resource Center cohort^61^. The 803 individuals in HBTRC cohort comprise 388 AD cases, 220 Huntington’s disease cases and 195 controls matched for age, gender, and post-mortem interval (PMI). The tissue specimens were provided by the HBTRC.

Epistasis was detected using a linear modelling approach, as implemented in the FastEpistasis software^62^. Briefly, to test for the presence of an epistatic effect of two SNPs on a phenotype, the relation between the value of the trait and the allele dosages at these two loci was modelled as the combination of separate additive effects and of a multiplicative effect. The model was fitted by linear regression, and the presence of epistasis assessed based on the F test p-value of the coefficient for the multiplicative term, relative to the hypothesis of a null effect. The significance of each interacting SNP pair is characterized by p-value < 1*e*^−8^ for the association with changes related to ventricular volume and Braak score and p-value < 1*e*^−5^ for the association with cognitive traits. The computation was run in parallel at the HPC center of the Vital-IT group at SIB, using software versions optimized to the diverse processor (intel(R) Xeon(R)) architectures and parallelization paradigms (OpenMP, MPI, Hybrid) available in this compute cluster.

Mapping of SNPs to genic and intergenic bins SNP IDs, for which genotype data were available, were mapped on genomic regions using the Ensembl database accessed via R package biomaRt ver. 2.28.0^63^. To make complete use of these genome-wide genotypes, we considered not only regions where genes are located, but also intergenic regions (IGR). The SNPs located within the boundaries of a gene, including a margin of 5k base-pairs on each side, were assigned to this gene and mapped to the corresponding ENSG ID. The SNPs located outside of these boundaries were assigned to the intergenic region delimited by two flanking genes. Each of these IGRs was uniquely identified by the combination of the ENSG IDs of the flanking genes. In this study we refer epistatic interactions that contain an IGR as inter genic region interactions (IGRI). Consult Online-only Table 1 to see the data sets containing IGRI. As genes may overlap, a single SNP may be assigned to multiple genes. Conversely, it can only be assigned to a single intergenic region. In the following, we refer to both gene regions and intergenic regions as “bins”. A region-wise Bonferroni correction method was used to adjust for multiple testing. The critical significance level alpha was adjusted separately for each pair of bins considered, using as the correction coefficient the number of pairs formed by the combination of SNPs located in the two bins.

Epistatic interactions were added to the HENA data set in the form of interaction between two nodes, node 1–node 2, with the corresponding ENSG.A and ENSG.B and a score, representing a p-value of an interaction. In the case of IGRI, the interacting nodes can be represented, for example, as ENSG.C–ENSG.D for node1 and ENSG.E–ENSG.F for node2, respectively, whereas ENSG.C, ENSG.D, ENSG.E, and ENSG.F are the flanking genes of the intergenic region.

### Yeast two-hybrid data generation and analysis

The yeast two-hybrid PPI data set of proteins related to brain ageing was produced by the Aged Brain SYSBIO consortium^64^. Studying the interactions between the proteins can reveal their function, and provide information about biological processes they participate in, including neurodegenerative diseases like Alzheimer’s disease.

All the baits were cloned using standard procedure after PCR amplification and the validity of the sequence was checked by full sequencing. The baits were used to screen a random-primed cDNA library constructed into the pP6. Human Brain (HBR) cDNA library. The prey fragments of the positive clones were amplified by PCR and sequenced at their 5′ and 3′ junctions. The resulting sequences were used to identify the corresponding interacting proteins in the GenBank database (NCBI) using a fully automated procedure. A confidence score (PBS, for Predicted Biological Score) was attributed to each interaction as previously described^65^. The protein interactions from this publication have been submitted to the IMEx consortium^66^ through IntAct^8^ and assigned the identifier IM-26801. Protein identifiers from the resulting PPI data set were mapped onto ENSG IDs using R package gProfileR^36^. The corresponding interactions were added to HENA data set in a form of interaction between two nodes ENSG.A–ENSG.B and a score representing PBS (please see Online-only Table 1 for details).

### Filtering of the protein-protein interactions from IntAct data sets

Three data sets of human protein-protein interactions were downloaded from the IntAct database version 4.2.6^8 ^– a human interaction data set^67^, a data set of expert-curated PPIs based on proteins with an association to Alzheimer disease (http://www.ebi.ac.uk/intact/query/annot:%22dataset:alzheimers%22), and a data set of PPI based on proteins with an established role in the presynapse (http://www.ebi.ac.uk/intact/query/annot:%22dataset:synapse%22). To keep interactions with medium or high confidence, as suggested by IntAct domain experts, we kept the data with the IntAct MI score ≥ 0.45. Data sets for human interactions were selected based on the following search criteria “(taxid: 9606 (human)) AND intact-miscore: [0.45 TO 1]”. Since the data sets also contained some interactions between human proteins with proteins of other species, we additionally filtered curated data sets ADIA and SIA based on the human taxonomy ID 9606 to select human-specific interactions. Protein names from the original data sets were mapped to Ensembl ver.93 gene name space (ENSG ID) using R package gProfileR^36^. The interactions between protein.A and protein.B were added to the HENA data set in a form of interaction ENSG.A–ENSG.B with the corresponding MI score^68^ (see Online-only Table 1 for details).

### Combining individual interaction data sets

Individual data sets of co-expression, epistasis and protein-protein interactions in the form of edge lists, obtained as described in the previous Methods sections, were combined into one data set. This data set can be represented as a *heterogeneous graph*, with nodes representing genes, and edges representing biological relations between the genes. HENA data set network structure^18,19^ is illustrated as a graph in Fig. 3 and represented as an example edge list in Table 4.

**Table 4:** Example of the interactions representation as an edge list.

ENSG.A	ENSG.B	Score	Interaction type	Data set ID in HENA
ENSG00000129484	ENSG00000167972	0.0001	PPI	PBA
ENSG00000119917	ENSG00000119917	0.68	PPI	ADIA
ENSG00000022355	ENSG00000000005	0.7238095	co-expression	ABA_CA1
ENSG00000050767	ENSG00000070950	9.30682e-09	epistasis	TGEN
…	…	…	…	…

Additionally, each node in the HENA data set is characterized by a set of attributes (“HENA NODE ATTRIBUTES” part of HENA data collection deposited at figshare repository)^20^, such as gene name, biotype^69^, GWAS p-value, positive selection p-value and gene expression in the 231 brain region. The acquisition of the individual attributes and the process of combining these attributes are described in the sections below.

### Gene expression in the brain regions

The same preprocessed six whole-brain microarray data sets from the Allen Human Brain Atlas^41–46^ were used to compute the aggregated gene expression in the 231 brain region annotated in the meta data accompanying the microarrays. Expression values for each gene in samples related to the individual brain region in all six brains were aggregated in a form of Z-scores. We first calculated mean expression values for each probe across all samples per region in each of the six data sets. We then calculated Z-scores over mean gene expression values in each individual region. The probe IDs were mapped to Ensembl ver.93 gene name space (ENSG ID) using gProfileR R package ver. 0.6.2^36^. In cases where two or more probe sets were mapped onto the same ENSG ID, the probe with absolute the maximum Z-score was kept in the data set.

### LOAD GWAS analysis

International Genomics of Alzheimer’s Project (IGAP)^14^ is a large two-stage study based upon genome-wide association studies (GWAS) on individuals of European ancestry. In stage 1, IGAP used genotyped and imputed data on 7055881 single nucleotide polymorphisms (SNPs) to meta-analyse four previously-published GWAS data sets consisting of 17008 Alzheimer’s disease cases and 37154 controls (the European Alzheimer’s disease Initiative, the Alzheimer Disease Genetics Consortium, the Cohorts for Heart and Aging Research in Genomic Epidemiology consortium, the Genetic and Environmental Risk in AD consortium). In stage 2, 11632 SNPs were genotyped and tested for association in an independent set of 8572 Alzheimer’s disease cases and 11312 controls. Finally, a meta-analysis was performed combining results from stages 1 & 2^70^. The resulting GWAS p-values from meta analysis of stages 1 and 2 were used as one of the node attributes. We have filtered the data set and kept SNPs with p-value ≤ 0.05. Original SNP IDs were converted to Ensembl name space ver. 93 using biomaRt R package ver. 2.28.0^63^ and combined with the set of node attributes (for details see section Aggregation of the node attributes). Mapping of the multiple SNP IDs to the same ENSG ID resulted in multiple corresponding records in the resulting data set. At the step of combining node attributes, we kept the original SNP ID as one of the node attributes (see section Combining node attributes for details).

### Positive selection analysis

In order to characterize the evolutionary dynamics in genes that are associated with Alzheimer’s disease, we analyzed the SNP data from IGAP^14,70^. We first mapped the 500 most significant SNPs onto their encoded human genes. As many SNPs mapped to the same genes, we removed duplicates, yielding a list of 42 unique genes.

These genes were converted to ENSG IDs and 1 to 1 orthologs for each of the corresponding human genes were searched across 41 mammalian species described in the tree of mammalian species topology. When querying the Ensembl database, only genes with transcripts whose status is “known” and “coding” were retained. Only 36 of the genes matched these criteria. In cases for which more than one transcript was available per human gene, the longest one was retained. In cases where more than one transcript was available for each of the mammalian orthologs, the transcript with the highest^71^ score against the retained human transcript was chosen. This procedure resulted in 23 genes for which at least 4 orthologs were collected. Codon multiple sequence alignments (MSA) for each of these sets were computed by first aligning the translated protein sequences using MAFFT v7.182^72^ and then back-translating this MSA to nucleotide-based alignment.

We next searched for positive Darwinian selection in each orthologous group. Two types of tests were conducted, site test and site-branch test. Both tests were conducted using PAML version 4^73^. For each gene, a mammalian species topology based on current literature^74–77^ was pruned to include only the species for which Ensembl orthologs were found. Each such pruned topology, together with the codon MSA, were provided as input into the PAML program. Out of the 23 genes 14 were shown to evolve under a positive selection regime using the site test based on FDR-corrected p-value ≤ 0.05. This data set is available as “Positive Darwinian selection” part of HENA data collection deposited at figshare repository^20^.

We next tested whether each gene experienced positive selection only in the branch leading to Homo sapiens. To test this hypothesis, we used the site-branch test^72^. In this test, the alternative model allows some sites to experience positive selection only in the lineage leading to Homo sapiens. However, no support for human-specific positive selection using this test was found.

The positive selection p-values were added to the list of node attributes. The values of this attribute is available for the genes shown to evolve under a positive selection regime using the site test.

### Combining node attributes

Ensemble ver. 93 gene names corresponding to the nodes in the HENA data set, e.g. proteins, genes and SNPs, were extracted from all individual processed data sets (Fig. 3) as it was described in the previous sections. To ease the usage of the integrated data set, Ensembl gene identifiers were converted also to gene names. Corresponding gene biotype according to Ensembl classification^69^ was extracted for each node using biomaRt R package ver. 2.28.0. Nodes with pseudogene biotypes were filtered out. Additionally, the interactions containing these nodes were also removed from the HENA data set. Individual gene attributes, i.e. ENSG ID, gene name, biotype, SNP ID, GWAS p-value, positive selection p-value and expression in the 231 brain region, were merged based on ENSG ID resulting in a vector of 237 attributes for each node^20^ (see Table 2).

## Data Records

**Fig. 4:**
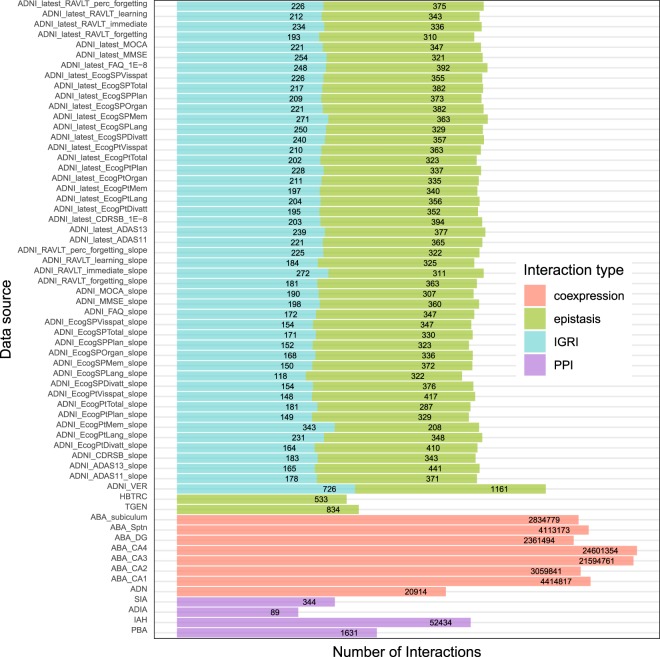
Counts of the interactions in the individual data sets comprising HENA data set. Each bar corresponds to the individual data set. Colors indicate interaction types: co-expression, PPI, epistasis ans IGRI. The number on top of the bar states the number of interactions.

This section describes data records originating from the preprocessed 64 individual data sets that comprise the HENA data set. For a compact description of the 46 data sets associated with cognitive traits, they are described in one data record 5. HENA data set consists of two parts, a graph, describing biological interactions (see Data records 1–10) between genes, proteins, SNPs, probes and a set of node attributes providing additional information about the genes (see Data record 11). A summary of the HENA data set is demonstrated in Fig. 4 and Online-only Table 1.

### Data record 1 - gene co-expression in AD and normal brain

Gene co-expression describes the correlation between changes in gene expression levels across multiple samples and biological conditions. The main idea is that two genes that have correlated expression across multiple biological conditions are more likely to encode interacting proteins^78^, i.e. proteins involved in the pathological disease processes. This data record represents a data set of gene pairs co-expressed in Alzheimer’s disease patients and healthy individuals. It can be downloaded as an individual data set “ADN precomputed gene co-expression in Alzheimer’s disease and healthy samples” from HENA data collection deposited at figshare repository^20^ or as a part of the integrated HENA data set^18,19^. Each co-expressed gene pair is described by a Robust Rank Aggregation (RRA) score^35^, ranging from 0 to 1 (the lower the score, the smaller the rank of the corresponding interaction). A summary of the attributes’ values associated with gene co-expression is shown in Online-only Table 1.

### Data record 2. Co-expression in disease-related brain regions

The development of AD pathology is not homogeneous across the brain. It has been shown that there are a number of brain regions such as hippocampal cornu ammonis sectors (CA1–CA4), dentate gyrus (DG), subiculum, and septal nuclei (SptN) which are affected by Alzheimer’s disease^1,49–54^. The pathological formation of amyloid-*β* plaques as well as the aggregation of the microtubule protein Tau forming neurofibrillary tangles lead to neuronal loss. Co-expressed gene pairs in these brain regions can help to identify the interacting proteins involved in the disease pathways. Co-expression in the disease-associated regions was computed using whole-brain microarray data sets downloaded from the Allen Brain Atlas^47^. The analysis resulted in seven individual co-expression data sets described in Online-only Table 1 and Fig. 2. These data sets can be downloaded as individual data sets “Co-expression in CA1 brain region”, “Co-expression in CA2 brain region”, “Co-expression in CA3 brain region”, “Co-expression in CA4 brain region, “Co-expression in DG brain region”, “Co-expression in subiculum brain region”, “Co-expression in SptN brain region” from HENA data collection available at figshare repository^20^ or as a part of the integrated HENA data set at NDEx repository^18,19^. Each co-expressed gene pair is characterized by a Spearman rank correlation coefficient. The association can be described as a positive or negative correlation. While a positive correlation can potentially indicate protein interaction and activation mechanism, a negative correlation will represent a scenario of the decrease in gene expression of one of the genes with the increase in the expression of its co-expressed partner leading to the suppression mechanism. The higher the absolute coefficient value, the stronger the co-expression between the genes is.

### Data record 3 - epistatic effects of pairs of SNPs on change in ventricle volume in ADNI cohort

Epistasis is an effect of interaction between two or more SNPs of different genes on a phenotype deviating from their individual effects^79,80^. These effects are especially interesting in the cases of complex traits such an Alzheimer’s disease. Change in ventricular volume detected on a patient’s MRI can serve as an indicator of brain tissue loss during the progression of dementia^81,82^. The data record represents epistatic effects of pairs of SNPs on change in ventricle volume detected in ADNI consortium^10^ patients (ADNI_VER). A summary of epistatic interactions in ADNI_VER is shown in Online-only Table 1. This data set is available for download as an individual data set “Epistasis Alzheimer’s Disease Neuroimaging Initiative (ADNI_VER)” from HENA data collection at figshare repository^20^ and as a part of the integrated data collection^18,19^.

### Data record 4 - epistatic interactions associated with Braak score in TGEN cohort

This data record is a data set of epistatic interactions between pairs of SNPs associated with Braak staging^57^ detected in the TGEN cohort (TGEN). Braak staging reflects the disease severity of Alzheimer’s disease based on pathophysiological changes in the brain.

A summary of the epistatic interactions in TGEN is shown in Online-only Table 1. The data set “Epistasis Translational Genomics Research Institute (TGEN)” is available for download individually from HENA data collection at figshare repository^20^ and as a part of the HENA integrated data set^18,19^.

### Data record 5 - epistatic interactions associated with Braak score in HBTRC cohort

This data record represents epistatic interactions between pairs of SNPs associated with Braak staging^57^ detected in the Harvard Brain Tissue Resource Center cohort (HBTRC). The summary of the HBTRC data set is depicted in Online-only Table 1. The data set “Epistasis Harvard Brain Tissue Resource Center (HBTRC)” is available for download individually from HENA data collection at figshare repository^20^ and as a part of the integrated HENA data set^18,19^.

### Data record 6 - epistasis interaction associated with cognitive traits in ADNI cohort

The disease’s progression can be described by a number of features such as changes in memory, attention and language. These features combined can characterize specific quantitative traits associated with the progression of dementia. This data record is comprised of 46 data sets of epistatic effects on 23 quantitative traits (see Table 3) of LOAD patients from the ADNI cohort. The summary of data sets and the corresponding data identifiers used in HENA are shown in Online-only Table 1. These 46 data sets are available for download in the form of a combined data set of epistatic effects on cognitive traits “Epistasis cognitive traits (ADNI_CT)” from HENA data collection at figshare repository^20^ and as a part of the integrated HENA data set^18,19^.

### Data record 7 - PPI involved in brain ageing

This data record describes newly characterized PPIs involved in brain ageing (PBA) including interactions between gene products from the late-onset Alzheimer’s disease genome-wide association study (LOAD-GWAS). The interactions were obtained using the yeast two–hybrid method. Each interaction is described by the predicted biological score (PBS). The PBS scores have been shown to positively correlate with the biological significance of the interactions^83,84^. A summary of the protein-protein interactions is shown in Online-only Table 1. Data are available for download as an individual data set^64^ and a preprocessed version as a part of the integrated data collection^18–20^.

### Data record 8 - PPI from IntAct in human

The data set contains information about medium and highly confident protein-protein interactions in humans that are available from the IntAct molecular interaction database^8^. The confidence level of each interaction is characterized by an IntAct MI score^68^; larger scores correspond to a higher confidence level. A summary of this data record is shown in Online-only Table 1. This data set can be downloaded as an individual fail “intact_int.txt” located under section “Preprocessed PPI data sets from IntAct included in HENA” of HENA data collection deposited at figshare repository^20^ and as a part of the integrated data collection^18,19^.

### Data record 9 - alzheimer’s disease PPI from IntAct

Alzheimer’s disease PPI from IntAct (ADIA) is a subset of the expert-curated PPI data set based on the proteins with an association to Alzheimer’s disease^85^ available from the IntAct molecular interaction database (http://www.ebi.ac.uk/intact/query/annot:%22dataset:alzheimers%22, 2017)^8^. The confidence level of each interaction is characterized by an IntAct MI score as described in Data record 8. Data were filtered as described in the Methods section. A summary of this data record is shown in Online-only Table 1. The filtered data set is available for download as a part of the integrated data collection^18,19^ and as an individual fail “alz_intact_int.txt” under “Preprocessed PPI data sets from IntAct included in HENA” section of HENA data collection deposited at Figshare repository^20^.

### Data record 10 - synaptic PPI from IntAct

The synaptic PPI data set from IntAct (SIA) contains automatically selected protein-protein interactions from the IntAct database^8^ with an established role in the presynapse (http://www.ebi.ac.uk/intact/query/annot:%22dataset:synapse%22, 2017). A selected set of interactions is comprised of protein pairs where at least one protein has an established link to the synapse. The confidence level of each interaction is characterized by an IntAct MI score as described in Data record 8. The data set was filtered as described in the Methods section. A summary of this data set attributes is depicted in Online-only Table 1. The filtered data set is available for download as a part of the integrated data collection^18,19^ and as an individual fail “syn_intact_int.txt” under “Preprocessed PPI data sets from IntAct included in HENA” section of HENA data collection deposited at figshare repository^20^.

### Data record 11 - aggregated information about the gene

The data record contains combined information about all nodes involved in the interactions described in the HENA data set^18,19^. Each node in this data set represents a gene, protein, SNP or probe that is uniquely identified by its Ensembl gene name, and is characterized by a set of attributes (Fig. 3 and Table 2). This data record combines preprocessed GWAS data set related to Alzheimer’s disease (“Preprocessed GWAS data included in HENA)^20^, a data set of genes showing positive selection (“Positive Darwinian selection”)^20^, and a data set of gene expression in brain regions (“Aggregated gene expression in six whole-brain microarray datasets”)^20^. Individual gene attributes, i.e. ENSG ID, gene name, biotype^69^, SNP ID, GWAS p-value, positive selection p-value and expression in the 231 brain region are combined based on ENSG ID and represent a vector of 237 attributes for each node (see Table 2). A summary of this data is presented in Online-only Table 1.

The data record also contains information about the association of 11,632 SNPs mapped onto ENSGs with Alzheimer’s disease^70^ based on a genome-wide association study performed by the International Genomics of Alzheimer’s Project^14^. Aggregated data about each gene also includes the information about positive Darwinian selection produced by the AgedBrainSYSBIO consortium (“Positive Darwinian selection” data set)^20^. Additionally, each gene in the data set is characterized by its biotype and gene name according to Ensembl database ver.93.

## Technical Validation

### Microarray gene expression preprocessing

The raw .CEL files and annotations of Affymetrix GeneChip Human Genome U133 Plus 2.0 microarray data sets^28–33^ were downloaded from ArrayExpress^7^ and preprocessed according to MEM standard operation pipeline^34^. The samples were quantile-normalised and background corrected using the just.rma() function from affy R package^86^ with default parameters. Preprocessed data sets were converted into NetCDF format using ncdf4 R package^87^. Probe set expression profiles in each normalized data set were filtered based on the standard deviation (SD) >0.29 to eliminate the probe sets with low variance. Additionally, only samples related to Alzheimer’s disease and healthy controls were selected for further analysis.

For the co-expression analysis in disease associated regions, six Agilent 8 × 60 K whole-brain gene expression microarrays^41–46^ were downloaded from the Allen Human Brain Atlas^47^. Data sets were normalized and background-corrected by data authors as described in technical white paper “Microarray data normalization^88^”. Additionally, combined data sets were quantile-normalised and filtered based on standard deviation to eliminate probes with low variance. Principle component analysis was applied to identify possible batch effects. The ontology of physiological brain structure that was used in the study is described in technical white paper “Ontology and nomenclature in the Allen Human Brain Atlas”^89^.

### Selecting the co-expression interactions

To ensure the selection of the reliable co-expression interactions, we have applied the following strategy. In the computations of the co-expression in disease-specific brain regions, in addition to the Spearman correlation coefficient, we have computed the significance level of each correlation using R package psych ver. 1.8.4^56^. The obtained p-values were FDR-corrected. Only interactions with p-value ≤ 0.01 were retained. Weakly co-expressed pairs were removed based on the distribution of the correlation coefficient. We kept the interactions with correlation values ≥1^st^ quartile of the positive correlation coefficient distribution and ≤3^rd^ quartile of the negative correlation coefficient distribution.

In the computations of co-expression in Alzheimer’s disease and non-disease samples, the resulting scores were reported in the form of an RRA^35^ score. These scores have been FDR-corrected and filtered based on a threshold of ≤1*e*^−5^ to ensure the reliability of the reported results.

### Genotyping and quality control

Genomic DNA samples were analyzed on different platforms in the three cohorts (ADNI, HBTRC, TGEN), as previously described in publications from respective institutions or consortiums.

TGEN cohort samples were genotyped on the Genome-Wide Human SNP 6.0 Array (Affymetrix, Inc., Santa Clara, CA, USA). Genotypes were called using Birdsuite. Standard quality control filters regarding minor allele frequency (MAF > 0.01), missing rate per marker and per individual (>0.15), and Hardy-Weinberg equilibrium (p < 1*e*^−6^) were applied using PLINK. The resulting data, available for 1599 subjects and 759916 markers, were downloaded in PLINK format from TGen^59^.

HBTRC cohort samples were genotyped on the Illumina HumanHap650Y array (Illumina, Inc., San Diego, CA, USA) by the Merck Research Laboratories. Genotype data (Version 01, 2011-01), available for 741 subjects and 555091 markers, were downloaded from the Sage Data Repository^61^. The Sage Data Repository dataset entry^61^ has been modified since the original dataset was downloaded at April 28^th^ 2015 from https://synapse.sagebase.org/#Synapse:syn4505. Thus we have stored the dataset used by us as part of the HENA data collection available via figshare repository^20^.

ADNI cohort samples were assayed using Human610-Quad BeadChip (Illumina, Inc., San Diego, CA, USA), which features a genome-wide selection of 620901 tag SNPs. GenomeStudio v2009.1 (Illumina) was used to generate SNP genotypes from bead intensity data^90^. The resulting data, available for 757 subjects, were downloaded in PLINK format from the ADNI repository^58^. As the two SNPs (rs429358, rs7412) that define the APOE epsilon alleles are not on the Human 610-Quad Bead Chip, APOE genotyping was performed separately, using restriction isotyping by gene amplification and cleavage with HhaI^91^. ADNI quality control steps included removing copy number variant probes as well as checking strand, base pair position, and allele specificity^92,93^.

### Filtering of individuals and markers

Additionally, SNPs were excluded according to the following criteria, call rate <0.1, minor allele frequency (MAF) <0.05, discrepancy relative to Hardy-Weinberg equilibrium (*p* ≤ 1*e*^−3^). Finally, tests for epistatic effects was limited to SNP pairs for which the product of the MAFs was greater than 0.01.

### Adjustment of Braak score phenotype

The Braak score is a semi-quantitative ordinal variable describing the extent of AD-characteristic alterations of neural tissue, from post-mortem examination of histological preparations. It defines six levels, from normal stage 1 to the most severe neuronal loss and accumulation of amyloid-beta plaques and neurofibrillary tangles corresponding to the stage 6. It is well known that Braak score increases with age, even in cognitively normal individuals. To adjust for this, the effect of age was estimated by loess regression, and subtracted from the observed score prior to analysis of epistatic effects.

### Adjustment of change in ventricular volume phenotype

Segmentation of cerebral ventricles and other brain structures on MRI images was performed at UCSF with the Freesurfer image analysis suite^94^, and the volumes reported in the ADNIMERGE data table^58^. In our study we have used the content of this table downloaded from the ADNI repository on January 30, 2014. The evolution of ventricular volume over time in each subject was characterized by the slope of a linear regression line fitted on the individual’s time-series of volume measurements, using the lm() function in R^95^, for a total of 621 subjects having at least two time-points, out of the 757 subjects with genotype data. To account for confounding effects of major covariates, a multiple linear model of the effects of age, gender and baseline disease status was fitted to the ventricle volume increase rates, using the lm() function in R^95^. The residual ventricle variation that remained unexplained by these covariates was used as the input for analysis of genetic epistatic interactions.

### Clone selection in yeast two-hybrid

Yeast two-hybrid screening was performed by Hybrigenics Services, S.A.S., Paris, France. The baits were fused to the C-terminal (LexA-bait, pB27) or the N-terminal (bait, lexA). The choice was made given the topology of the protein and knowledge available on functional fusion in mammalian cell. pB27, pB29 and pP6 were derived from the original pBTM116^96^ and pGADGH^97^ plasmids, respectively. For baits with very few positive clones in the less selective media, using the LexA-based plasmid, the same bait fragments have been sub-cloned in Gal4-based plasmids pB66 (Gal4-Bait) or pB43 (Bait-Gal4) and submitted to a new screen process pB66 and pB43 derive from pAS2DD^98^. For three baits (CELF1, FYN full length, MCF2L), the classic construct was too toxic to assure a saturated screening of the library and these were transferred into a centromeric met25 inducible vector and the induction of the bait was obtained in the selective media using minus methionine selective plates.

The screened Human Brain HBR cDNA library is a random primed library with a complexity of more than 50 million independent clones in *E. coli* and 10 million independent clones in yeast. The libraries were screened using a mating approach with YHGX13 (Y187 ade2-101::loxP-kanMX-loxP, mat *α*) containing the pre-transformed libraries and L40 ΔGal4 yeast strains containing the baits as previously described^98^. This method allows full coverage of the library. Potential auto-activation or stickiness of the bait was compensated by the use of 3-aminotriazole (3-AT). The number of selected clones by million diploids was maintained below 10 with a mean of 1.2. The selectivity of the screen was then maintained to limit the background.

### Selecting reliable PPI

The current data set includes a yeast two-hybrid data set generated by the consortium and three data sets from IntAct database^8^. The confidence of the PPI can be evaluated based on the corresponding score.

Yeast two-hybrid PPIs are characterized by a PBS score^83,84^. The PBS relies on two different levels of analysis. Firstly, a local score takes into account the redundancy and independence of prey fragments, as well as the distribution of reading frames and stop codons in the overlapping fragments. Secondly, a global score takes into account the interactions found in all the screens performed using the same library. This global score represents the probability of an interaction being nonspecific. The score ranges from 0 to 10 (the smaller the score, the more specific the interaction). In the HENA data set, we did not enforce any filtering on PBS, leaving the opportunity for the user to decide what threshold for the interactions is the most desirable.

Interactions from the IntAct database are characterized by the MI score. It is based on the manual annotation of every instance of a binary interaction within the IntAct database, taking into account information about the interaction detection method, interaction type, and the number of publications the interaction has appeared in. The cumulative score is normalized between 0 and 1 across the entire IntAct database, with 1 representing an interaction with the highest confidence. The interactions added to HENA were selected based on the medium to high confidence level corresponding to MI score range [0.45; 1].

### Detecting positively selected genes

Two types of tests were conducted to identify positively selected genes as described in the Methods section. The resulting p-values were adjusted for multiple comparisons using the FDR method. Genes evolving under a positive selection regime were chosen based on p-value ≤ 0.05.

### Comparison with public databases

**Fig. 5:**
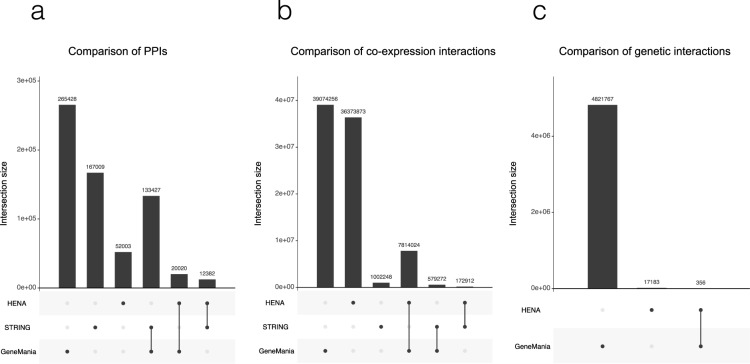
Comparison of HENA data set with public databases. The number of intersecting interactions between HENA, GeneMania and STRING are demonstrated respectively in, (**a**) PPI, (**b**) co-expression and (**c**) genetic interactions. The line between the two data sets in the figure meta-data displayed under x-axes represents the intersecting sets. In the case of a data set intersecting with itself, a single dot is displayed.

Although there is no alternative Alzheimer’s disease-specific database or data set such as HENA available to our knowledge, we have selected for the comparison two widely used general-purpose databases that use network structure as underlying architecture of the data representation. We have compared the Alzheimer’s disease-specific PPI, co-expression and epistatic interactions collected in HENA with the interactions of the matching type from STRING^99^ and GeneMANIA^100^. Comparisons were conducted for the pairs of interacting nodes. While GeneMania reports interactions using ENSG IDs, STRING uses Ensembl protein identifiers. For the sake of consistent comparison we have mapped Ensembl protein identifiers to ENSG IDs. It is not a trivial task to find a suitable data source for the comparison of epistatic interactions due to the lack of such resource. We have compared the epistatic interactions with genetic interactions from GeneMania solely as the STRING database does not contain similar interaction types. We have excluded IGRIs from the comparison due to the lack of similar types of interactions in these two public databases. All data sets were prepared for the comparison by keeping only binary undirected interactions in the form of ENSG.A-ENSG.B. The overlapping interactions of the same type were counted and visualized using UpSetR R package^101^ for the visualization of intersecting sets. The results of the comparison are displayed in Fig. 5.

Comparison of the computational data sets such as co-expression and epistasis is highly dependent on the conditions used to produce a particular data set. Therefore, substantial discrepancies are possible when comparing computational data sets obtained in different setups, i.e. disease- and phenotype-specific co-expression and epistatic interactions might differ from the interactions reported for other conditions. Additionally, the discrepancy between the number of overlapping interactions can be caused by the difference in how frequently data sources are being updated and criteria for the interactions to be included into a database. For example, in the case of the PPI comparison we found out examples of PPIs, such as MAD1L1 - LMO3 with a MI score of 0.49, that is considered to be interaction with medium-high confidence by IntAct, but they were not present in STRING database as was expected because STRING includes interactions from IntAct. There are a few reasons why interactions may not appear in STRING. Firstly, STRING is updated every two years. Secondly, the data sets imported to STRING are re-benchmarked for each interaction A-B, taking into account the number of cliques and the number of interacting partners for both proteins A and B in the particular data set.

## Usage Notes

The HENA data set^18^ is available from the NDEx repository^17^ which allows convenient sharing and exchange of network data sets. Due to the difficulty of incorporating the nodes representing IGRs, we have omitted them from the data set shared via the NDEx repository^18,19^. However, HENA with IGR nodes is available from the figshare repository^20^ in a tab-separated format. Besides full version of HENA^18^, we have created a reduced version^19^ that sets a much more restrictive threshold (co-expression coefficient ≤−0.8 and ≥0.8) for the inclusion of co-expression edges described in Data record 2. This reduced version of HENA^19^ is aimed to be used by a biologist in simple operations such as exploring a network neighborhood around genes of interest. Both versions of HENA can be viewed and queried based on the filters available at the web user interface of NDEx repository, and further opened and manipulated in Cytoscape^24^ as described in the NDEx user manual^102^.

The expanding volume and variety of data across different biological domains allows researchers to analyze these diverse data and integrate them with their own work (Figs 1 and 2), e.g. to propose new hypotheses and find answers to the biological questions of interest related to Alzheimer’s disease. However, novel machine-learning algorithms are needed to utilize such heterogeneous big data. Below we demonstrate how HENA can be analysed using graph convolutional networks, a state-of-the-art deep learning methods for large graphs^23,103,104^.

### Analysis of the heterogeneous graph using graph convolutional networks

The understanding of disease mechanisms on different levels is not a trivial task. Here we demonstrate an application of state-of-the art graph convolutional networks to identify genes that are potentially associated with Alzheimer’s disease using biological information about genes and interactions of different types between pairs of genes and proteins. We also show how additional data sets can be used together with the HENA data set.

The most straightforward way to approach this problem would be to use a supervised machine learning approach, where genes are labeled based on their association with Alzheimer’s disease. Using the labeled set of genes we can train a model to find a decision boundary between two classes, and apply it to predict the association for the rest of the genes.

#### Combining HENA with an additional data set

Defining a positive and negative class for the model: Despite the substantial number of studies that have been carried out in the field of Alzheimer’s disease research, a set of confirmed positive and negative associations of genes and Alzheimer’s disease is not yet well defined. The genes (and proteins as their products) can be defined as associated with the disease, for example, based on genome-wide association studies and based on domain-expert-curated knowledge. We used information about the nodes from HENA to assemble a set of 944 genes associated with the disease based on the GWAS data set and Alzheimer’s-related PPI data set collected in HENA. Selecting a set of genes that is clearly not associated with the Alzheimer’s disease is even more challenging, and leads to the difficulty of defining the negative class. In this case study we approached this challenge by defining a negative class as a set of 1572 essential non-disease genes described in the evolutionary study by Spataro *et al*.^105^. The rest of the genes present in HENA are labeled as unknown.

Heterogeneous graph as an input for the model: The HENA data set can be represented as a *heterogeneous graph*, with nodes representing genes, and edges representing multiple biological relations between the genes (see Fig. 3). For this case study we have excluded IGRI to keep interactions between nodes that were mapped directly to genes, and co-expression interactions in disease related brain regions with low co-expression values (<0.5). Some sets of node attributes (or features) in HENA are incomplete due to the absence of the required information in the various sources describing them. We have also excluded the feature describing gene positive selection since it was not present for most of the genes. Nodes in the network were labeled according to association of the corresponding genes to Alzheimer’s disease, i.e. positive/negative labels for those known to be associated/not associated with the disease, unknown for those whose association with the disease is unknown. A summary of the resulting graph is shown in Table 5.

**Table 5:** Number of nodes and edges for each subgraph.

	# Nodes	# Edges
full graph	24825	9740721
PPI subgraph	10445	52003
co-expression subgraph	14634	9671535
epistasis subgraph	13881	17183

#### Graph convolutional networks for heterogeneous graphs

Generalization of GCNs for heterogeneous graphs: Recent advances in network analysis demonstrate that methods based on graph convolutional networks (GCN) outperform other traditional methods for many classification tasks on network data^106^. One of the GCN-related approaches, the GraphSAGE model, was proposed by Hamilton *et al*.^23^. It addresses several major issues that other GCN approaches suffer from. The most relevant to this study is scalability — most of the methods can not be applied to large graphs, while GraphSAGE is scalable to graphs with billions of nodes^107^. The second issue is the ability to work in an inductive setting as opposed to a transductive one. In a nutshell, the transductive learning does not generalize to unseen nodes, while inductive GraphSAGE can generate node embeddings — node feature vector representations — for nodes that were not seen during the training. We apply a generalization of the GraphSAGE algorithm^23^ to heterogeneous networks — HinSAGE^103^. The main difference from the homogeneous GraphSAGE^23^ is that HinSAGE takes into account and creates embeddings for different edge types.

Feature generation: To determine whether network topological features can provide additional information for the model, we have created three sets of features — *biological features*, *graph-related features* and a combined set of biological and graph-related features that we refer as *all features*.

Biological features here represent levels of aggregated gene expression in the 231 brain region and a value, representing genes, expressed higher in disease-associated regions (CA1-CA4, DG, SptN, subiculum) compared with the rest of the brain regions. This value was obtained by the application of the Wilcoxon test^108^ to compare gene expression in disease-associated regions and the rest of the brain regions.

Graph features for each node represent a combination of graph embeddings, generated by GraphSAGE^23^ and proportions of Alzheimer’s disease-associated genes in the first and second hop neighbourhood of each node. In this study we use an unsupervised version of GraphSAGE, where for each node we learn embeddings, i.e. low-dimensional vector representations for network nodes, based on both node features and the graph structure. Each node is then represented via a numeric vector of a specified fixed dimension (256 in our case) that captures the node properties in the graph in addition to the node features. Moreover, as we deal with a heterogeneous graph, we learn embeddings for each of the three sub-graphs, where a subgraph consists of edges of a particular type, i.e. co-expression, PPI, epistasis. Therefore, the dimensionality of the resulting embedding vector for each node is 256 × 3 as we concatenate embeddings from sub-graphs for each edge type.

A third set of features *all features* consists of the combined biological features and graph-related features.

Nodes with these three sets of features are then used as an input for the classification model HinSAGE. The performance of the model on three different feature sets is described in Table 6.

**Table 6:** Comparison of the model performance for different feature sets and for two models.

	HinSAGE			
	Precision	Recall	F1 score	ROC AUC
All	0.40	0.98	0.57	0.60
Graph	0.40	0.98	0.57	0.66
Biological	0.37	0.94	0.54	0.56
	**Random Forest**			
All	0.50	0.50	0.50	0.82
Graph	0.50	0.50	0.50	0.83
Biological	0.43	0.27	0.33	0.56

Comparison with the classical supervised model: Next, we adopt a classical supervised strategy, where a random forest classifier^104^ that is trained on the set of positive and negative genes for each of the feature sets. The results of both models’ performances are shown in Table 6. We then apply the models to the set of unknown cases and rank them according to the probability score. The higher the probability, the more likely the gene is associated with Alzheimer’s disease.

We have compared the results of random forest and HinSAGE model performance for biological, graph-related, and joined set of features. The results demonstrate that the model improves with the use of graph features. Random forest on biological features fails to get any information, all Alzheimer genes are classified as non-Alzheimer. HinSAGE on biological features uses the initially provided non-graph features, and propagates this information exploiting the graph structure. It demonstrates better performance than random forest on a set of biological features. We can notice that the performance of both models improves with the introduction of graph features. It is reflected by all indicated model performance metrics, i.e. precision, recall, F1, ROC AUC. However, due to positive and negative class imbalance, we refer to the F1 score, i.e. a harmonic average of the precision and recall of the model, as the most representative metric of model performance. Taking this into account, we conclude that Random Forest on graph-related feature set improves the performance, while HinSAGE on graph features is the best performing method.

#### Graph structure helps to capture complex relationships

For the exploratory analysis we selected genes suggested as candidates for an association with the Alzheimer’s disease by the algorithms. Here we performed surveyed the existing body of research about the suggested genes.

For this purpose we have created a list of genes, shown to have a strong association with the disease, from the recent publications. These publications include GWAS^109–111^ and genome-wide association study by proxy (GWAX) studies^112^, list of disease-specific autoantibodies in human sera^113^, list of genes reported to be associated in Alzheimer’s disease downloaded from the MalaCards database^114^, and the results of integrative transcriptome analysis by Raj *et al*.^115^. This combined list resulted in 169 nodes present in the result of the classification. For each model results we have selected genes that were assigned a probability of ≥0.5 to be associated with Alzheimer’s disease. Random forest classified 14 out if 169 genes to be associated with the disease while HinSAGE classified 154 genes from the list to be associated with the disease. Interestingly, the genes ACE, ADAMTS4 and CLNK, recently reported in three independent GWAS meta analysis publications^109–111^ were classified as Alzheimer’s disease-related genes with the corresponding probabilities 0.81, 0.93 and 0.88 while random forest did not classify them as Alzheimer’s disease-related genes.

These qualitative findings of the subset of genes demonstrate that graph structure is a rich data source that helps to capture complex relationships and find the distinctive patterns that are not easily detectable otherwise.

The prediction of genes related to complex diseases, such as Alzheimer’s disease, is not a trivial task. The ambiguity in the definition of the node class, i.e. relation to the disease, and selection of the informative features strongly influences the model performance. However, we have demonstrated the advantage of using graph structural information in node classification task compared to using biologically determined features alone.

### ISA-Tab metadata file

Download metadata file

## Data Availability

Data integration and analysis were performed using R language for statistical computing^95^ version 3.0.2 (2013-05-16), R version 3.3.1 (2016-06-21), R version 3.4.2 (2017-09-18). The case study was built using Python 3.6. The project repository is accessible via GitHub HENA repository and accompanied by DOI^116,117^.

## Online-only Table

**Online-only Table 1:** Summary of the individual data sets comprising HENA data set.

Original source	Datarecord	Data source in HENA	Interaction type	Score range	# Nodes	# Interactions
MEM	#1	ADN	co-expression	(0; 1.0e-05]	5559	20914
ABA	#2	ABA_CA1	co-expression	[−0.95; −0.24] [0.3, 1]	10537	4414817
		ABA_CA2			10537	3059841
		ABA_CA3			10537	21594761
		ABA_CA4			10537	24601354
		ABA_DG			10222	2361494
		ABA_SptN			10537	4113173
		ABA_subiculum			10537	2834779
ADNI	#3	ADNI_VER	epistasis, IGRI	(0; 1.0e-08]	1249	1887
TGEN	#4	TGEN	epistasis	(0; 1.0e-08]	1177	834
HBTRC	#5	HBTRC	epistasis	(0; 1.0e-08]	774	533
ADNI	#6	ADNI_ADAS11_slope	epistasis, IGRI	(0; 1.0e-05]	715	549
		ADNI_latest_ADAS11			915	586
		ADNI_ADAS13_slope			684	606
		ADNI_latest_ADAS13			975	616
		ADNI_CDRSB_slope			528	526
		ADNI_latest_CDRSB_1E-8			907	597
		ADNI_EcogPtDivatt_slope			698	574
		ADNI_latest_EcogPtDivatt			754	547
		ADNI_EcogPtLang_slope			652	579
		ADNI_latest_EcogPtLang			862	560
		ADNI_EcogPtMem_slope			647	551
		ADNI_latest_EcogPtMem			815	537
		ADNI_EcogPtPlan_slope			581	478
		ADNI_latest_EcogPtPlan			798	565
		ADNI_EcogPtTotal_slope			596	468
		ADNI_latest_EcogPtTotal			789	525
		ADNI_EcogPtVisspat_slope			589	565
		ADNI_latest_EcogPtVisspat			793	573
		ADNI_EcogSPDivatt_slope			569	530
		ADNI_latest_EcogSPDivatt			1002	597
		ADNI_EcogSPLang_slope			607	440
		ADNI_latest_EcogSPLang			934	579
		ADNI_EcogSPMem_slope			667	522
		ADNI_latest_EcogSPMem			1010	634
		ADNI_EcogSPOrgan_slope			671	504
		ADNI_latest_EcogSPOrgan			978	603
		ADNI_EcogSPPlan_slope			650	475
		ADNI_latest_EcogSPPlan			913	582
		ADNI_EcogSPTotal_slope			713	501
		ADNI_latest_EcogSPTotal			954	599
		ADNI_EcogSPVisspat_slope			647	501
		ADNI_latest_EcogSPVisspat			870	581
		ADNI_FAQ_slope			551	519
		ADNI_latest_FAQ_1E-8			1006	640
		ADNI_MMSE_slope			606	558
		ADNI_latest_MMSE			918	575
		ADNI_MOCA_slope			647	497
		ADNI_latest_MOCA			883	568
		ADNI_RAVLT_forgetting_slope			568	544
		ADNI_latets_RAVLT_forgetting			719	503
		ADNI_RAVLT_immediate_slope			568	544
		ADNI_latest_RAVLT_immediate			918	570
		ADNI_RAVLT_learning_slope			509	583
		ADNI_latest_RAVLT_learning			897	555
		ADNI_RAVLT_perc_forgetting_slope			537	547
		ADNI_latest_RAVLT_perc_forgetting			752	601
PBA	#7	PBA	PPI	[0.001; 10]	880	1631
IntAct	#8	IAH	PPI	[0.45; 1]	10142	52449
IntAct	#9	ADIA	PPI	[0.45; 1]	80	89
IntAct	#10	SIA	PPI	[0.45; 1]	315	344
GWAS	#11	Node attributes	NA	[0; 0.05]	1332	NA
PS				[0; 0.05]	14	
ABA				[−1.87; 2.07]	17803	
ENSEMBL				for regular nodes:esng ID,gene name,biotype;	28430	
				or IGR nodes:ensg1-ensg2,g.name1-g.name2, bio.t1-bio.t2	4352	

## References

[1] Masters, C. Alzheimer’s disease. *Nat*. *Rev*. *Dis*. *Prim*. **1,** 1–18 (2015). 10.1038/nrdp.2015.56 27188934

[2] Blennow K (2010). Biomarkers in Alzheimer’s disease drug development. Nat. Med..

[3] Hokama M (2014). Altered expression of diabetes-related genes in Alzheimer’s disease brains: the Hisayama study. Cereb. Cortex..

[4] Guerreiro R (2013). TREM2 variants in Alzheimer’s disease. New Engl. J. Med..

[5] Heneka M (2015). Neuroinflammation in Alzheimer’s disease. Lancet Neurol..

[6] Liang W (2008). Alzheimer’s disease is associated with reduced expression of energy metabolism genes in posterior cingulate neurons. Proc. Natl. Acad. Sci. USA.

[7] Brazma A (2003). ArrayExpress - a public repository for microarray gene expression data at the EBI. Nucleic Acids Res..

[8] Orchard S (2013). The MIntAct project—IntAct as a common curation platform for 11 molecular interaction databases. Nucleic Acids Res..

[9] Drew K (2017). Integration of over 9,000 mass spectrometry experiments builds a global map of human protein complexes. Mol. Syst. Biol..

[10] Petersen R (2010). Alzheimer’s disease Neuroimaging Initiative (ADNI) clinical characterization. Neurology.

[11] Zhang B (2013). Integrated systems approach identifies genetic nodes and networks in late-onset Alzheimer’s disease. Cell.

[12] Bennett D, Yu L, Dejager P (2014). Building a pipeline to discover and validate novel therapeutic targets and lead compounds for Alzheimer’s disease. Biochem. Pharmacol..

[13] Saykin A (2015). Genetic studies of quantitative MCI and AD phenotypes in ADNI: Progress, opportunities, and plans. Alzheimers Dement..

[14] Lambert J (2013). Meta-analysis of 74,046 individuals identifies 11 new susceptibility loci for Alzheimer’s disease. Nat. Genet..

[15] Jack C (2013). Tracking pathophysiological processes in Alzheimer’s disease: an updated hypothetical model of dynamic biomarkers. Lancet Neurol..

[16] Bateman R (2012). Clinical and biomarker changes in dominantly inherited Alzheimer’s disease. N. Engl. J. Med..

[17] Pratt D (2015). NDEx, the network data exchange. Cell Syst..

[18] Sugis E (2019). The Network Data Exchange (NDEx).

[19] Sugis E (2019). The Network Data Exchange (NDEx).

[20] Sugis E (2019). Figshare.

[21] AgedBrainSYSBIO consortium, http://agedbrainsysbio.eu/ (2017).

[22] Ritchie M, Holzinger E, Li R, Pendergrass S, Kim D (2015). Methods of integrating data to uncover genotype–phenotype interactions. Nat. Rev. Genet..

[23] Hamilton, W., Ying, Z. & Leskovec, J. Inductive representation learning on large graphs. *Adv*. *Neur*. *In*. **31**, 1024–1034 (2017).

[24] Shannon P (2003). Cytoscape: a software environment for integrated models of biomolecular interaction networks. Genome Res..

[25] Agrawal M, Zitnik M, Leskovec J (2018). Large-scale analysis of disease pathways in the human interactome. Pac. Symp. Biocomput..

[26] Lapatas V, Stefanidakis M, Jimenez R, Via A, Schneider M (2015). Data integration in biological research: an overview. J. Biol. Res-Thessalon..

[27] Zerbino D (2017). Ensembl 2018. Nucleic Acids Res..

[28] (2010). Array Express.

[29] (2010). Array Express.

[30] (2010). Array Express.

[31] (2010). Array Express.

[32] (2010). Array Express.

[33] (2010). Array Express.

[34] Adler P (2009). Mining for coexpression across hundreds of datasets using novel rank aggregation and visualization methods. Genome Biol..

[35] Kolde R, Laur S, Adler P, Vilo J (2012). Robust rank aggregation for gene list integration and meta-analysis. Bioinformatics.

[36] Reimand, J. & Kolde, R. Arak. *gPprofiler: Interface to the “g: Profiler” toolkit*. R package version 0.6.2, https://CRAN.R-project.org/package=gProfileR (2016).

[37] Microsoft Corporation and Steve Weston, doParallel: Foreach Parallel Adaptor for the ‘parallel’ R Package. R package version 1.0.14, https://CRAN.R-project.org/package=doParallel (2018).

[38] Ge H, Liu Z, Church G, Vidal M (2001). Correlation between transcriptome and interactome mapping data from Saccharomyces cerevisiae. Nat. Genet..

[39] Kemmeren P (2002). Protein interaction verification and functional annotation by integrated analysis of genome-scale data. Mol. Cell.

[40] Wolfe C, Kohane I, Butte A (2005). Systematic survey reveals general applicability of “guilt-by-association” within gene coexpression networks. Bmc Bioinformatics.

[41] (2013). Allen Brain Atlas.

[42] (2013). Allen Brain Atlas.

[43] (2013). Allen Brain Atlas.

[44] (2013). Allen Brain Atlas.

[45] (2013). Allen Brain Atlas.

[46] (2013). Allen Brain Atlas.

[47] Hawrylycz M (2012). An anatomically comprehensive atlas of the adult human brain transcriptome. Nature.

[48] Webb, A. *Statistical pattern recognition*. Ch. 2 (John Wiley & Sons Ltd, 2002).

[49] Selkoe D (2002). Alzheimer’s disease is a synaptic failure. Science.

[50] De Calignon A (2012). Propagation of tau pathology in a model of early Alzheimer’s disease. Neuron.

[51] Mueller S (2010). Hippocampal atrophy patterns in mild cognitive impairment and Alzheimer’s disease. Hum. Brain Mapp..

[52] Kim J (2015). Proteome-wide characterization of signalling interactions in the hippocampal CA4/DG subfield of patients with Alzheimer’s disease. Sci. Rep..

[53] Gan C, O’sullivan M, Metzler-Baddeley C, Halpin S (2017). Association of imaging abnormalities of the subcallosal septal area with Alzheimer’s disease and mild cognitive impairment. Clin. Radiol..

[54] Nicholson R (2010). Regional cerebral glucose uptake in the 3xTG model of Alzheimer’s disease highlights common regional vulnerability across AD mouse models. Brain Res..

[55] Allen Institute for Brain Science, Allen Human Brain Atlas. Technical White Paper: Ontology and nomenclature in the Allen Human Brain Atlas, http://help.brain-map.org/display/humanbrain/Documentation (2013).

[56] William Revelle psych: Procedures for Psychological, Psychometric, and Personality Research. R package version 1.8.4 (2018).

[57] Braak H, Braak E (1991). Neuropathological stageing of Alzheimer-related changes. Acta Neuropathol..

[58] Alzheimer’s Disease Neuroimaging Initiative: ADNI. *ADNI*, http://adni.loni.usc.edu/ (2015).

[59] The Translational Genomics Research Institute. *TGEN*, https://www.tgen.org/ (2015).

[60] Corneveaux J (2010). Association of CR1, CLU and PICALM with Alzheimer’s disease in a cohort of clinically characterized and neuropathologically verified individuals. Hum. Mol. Genet..

[61] Zhang, B. & Gaiteri, C. The Harvard Brain Tissue Resource Center (HBTRC) study. *Synapse*, https://www.synapse.org/#!Synapse:syn3159435 (2015).

[62] Schüpbach T, Xenarios I, Bergmann S, Kapur K (2010). FastEpistasis: a high performance computing solution for quantitative trait epistasis. Bioinformatics.

[63] Durinck S, Spellman P, Birney E, Huber W (2009). Mapping identifiers for the integration of genomic datasets with the R/Bioconductor package biomaRt. Nature Protocols.

[64] (2019). IntAct.

[65] Formstecher E (2005). Protein interaction mapping: a Drosophila case study. Genome Res..

[66] Embl-Ebi IMEx data. *IMEX*, https://www.imexconsortium.org/ (2019).

[67] Embl-Ebi. *IntAct*, http://www.ebi.ac.uk/intact/ (2017).

[68] Villaveces J. M., Jimenez R. C., Porras P., del-Toro N., Duesbury M., Dumousseau M., Orchard S., Choi H., Ping P., Zong N. C., Askenazi M., Habermann B. H., Hermjakob H. (2015). Merging and scoring molecular interactions utilising existing community standards: tools, use-cases and a case study. Database.

[69] Ensembl database, Gene and transcript types, https://www.ensembl.org/info/genome/genebuild/biotypes.html (2018).

[70] International Genomics of Alzheimer’s Project (IGAP) GWAS analysis stage 1&2. *IGAP*, http://web.pasteur-lille.fr/en/recherche/u744/igap/igap_download.php (2013).

[71] Needleman S, Wunsch C (1970). A general method applicable to the search for similarities in the amino acid sequence of two proteins. J. Mol. Biol..

[72] Katoh, K. & Ley, D. MAFFT: iterative refinement and additional methods. *Methods Mol*. *Biol*. **1079**, 131–146 (2014). 10.1007/978-1-62703-646-7_8 24170399

[73] Yang Z (2007). PAML 4: phylogenetic analysis by maximum likelihood. Mol. Biol. Evol..

[74] Blanga-Kanfi S (2009). Rodent phylogeny revised: analysis of six nuclear genes from all major rodent clades. BMC Evol. Biol..

[75] Perelman P (2011). A molecular phylogeny of living primates. Plos Genet..

[76] Nyakatura K, Bininda-Emonds O (2012). Updating the evolutionary history of Carnivora (Mammalia): a new species-level supertree complete with divergence time estimates. BMC Biol..

[77] Song S, Liu L, Edwards S, Wu S (2012). Resolving conflict in eutherian mammal phylogeny using phylogenomics and the multispecies coalescent model. Proc. Natl. Acad. Sci. USA.

[78] Snider J (2015). Fundamentals of protein interaction network mapping. Mol. Syst. Biol..

[79] Cordell H (2002). Epistasis: what it means, what it doesn’t mean, and statistical methods to detect it in humans. Hum. Mol. Genet..

[80] Phillips P (2008). Epistasis - the essential role of gene interactions in the structure and evolution of genetic systems. Nat. Rev. Genet..

[81] Madsen, S. *et al*. Mapping dynamic changes in ventricular volume onto baseline cortical surfaces in normal aging, mci, and Alzheimer’s disease. *Multimodal Brain Image Anal*. **8159**, 84–94 (2013). 10.1007/978-3-319-02126-3_9 PMC4138607 25152934

[82] Carmichael O (2007). Ventricular volume and dementia progression in the Cardiovascular Health Study. Neurobiol. Aging.

[83] Rain J (2001). The protein–protein interaction map of Helicobacter pylori. Nature.

[84] Wojcik J, Boneca I, Legrain P (2002). Prediction, assessment and validation of protein interaction maps in bacteria. J. Mol. Biol..

[85] Perreau V (2010). A domain level interaction network of amyloid precursor protein and A*β* of Alzheimer’s disease. Proteomics.

[86] Gautier L, Cope L, Bolstad B, Irizarry R (2004). Affy – analysis of Affymetrix Gene Chip data at the probe level. Bioinformatics.

[87] Pierce, D. ncdf4: Interface to Unidata netCDF (version 4 or earlier) format data files. R Package, http://cran.R-project.Org/package=Ncdf4 (2012).

[88] Human Allen Brain Atlas. Technical white paper: Microarray data normalization, http://help.brain-map.org/download/attachments/2818165/Normalization_WhitePaper.pdf?version=1modificationDate=1361836502191 api=v2 (2013).

[89] Human Allen Brain Atlas. Thechnical white paper: Ontology and nomenclature in the Allen Human Brain Atlas, http://help.brain-map.org/download/attachments/2818165/HBA_Ontology-and-Nomenclature.pdf?version=1modificationDate=1382051847989 api=v2 (2013).

[90] Saykin A (2010). Alzheimer’s Disease Neuroimaging Initiative biomarkers as quantitative phenotypes: genetics core aims, progress, and plans. Alzheimers Dement..

[91] Hixson J, Vernier D (1990). Restriction isotyping of human apolipoprotein E by gene amplification and cleavage with HhaI. J. Lipid Res..

[92] Biffi A (2010). Genetic variation and neuroimaging measures in Alzheimer disease. Arch. Neurol..

[93] Koran M, Hohman T, Meda S, Thornton-Wells T (2014). Genetic interactions within inositol-related pathways are associated with longitudinal changes in ventricle size. J. Alzheimers Dis..

[94] Fischl B (2012). Free Surfer. Neuroimage.

[95] R Core Team. R: A language and environment for statistical computing, R Foundation for Statistical Computing, Vienna, Austria. http://www.R-project.org/ (2015).

[96] Vojtek A, Hollenberg S (1995). Ras-Raf interaction: two-hybrid analysis. Methods Enzymol..

[97] Bartel, P. Using the two-hybrid system to detect protein-protein interactions. *Cellular Interactions In Development: Practical Approach* **254**, 153–179 (1993).

[98] Fromont-Racine M, Rain J, Legrain P (1997). Toward a functional analysis of the yeast genome through exhaustive two-hybrid screens. Nat. Genet..

[99] Szklarczyk Damian, Morris John H, Cook Helen, Kuhn Michael, Wyder Stefan, Simonovic Milan, Santos Alberto, Doncheva Nadezhda T, Roth Alexander, Bork Peer, Jensen Lars J., von Mering Christian (2016). The STRING database in 2017: quality-controlled protein–protein association networks, made broadly accessible. Nucleic Acids Research.

[100] Warde-Farley D (2010). The GeneMANIA prediction server: biological network integration for gene prioritization and predicting gene function. Nucleic Acids Res..

[101] Conway J, Lex A, Gehlenborg N (2017). UpSetR: an R package for the visualization of intersecting sets and their properties. Bioinformatics.

[102] The NDEx Project, Finding and Querying Networks, http://www.home.ndexbio.org/finding-and-querying-networks/ (2018).

[103] CSIRO data 61 investigative analytics, Stellar-ml v0.2.0: Machine Learning on graphs, https://github.com/stellargraph (2018).

[104] Breiman L (2001). Random forests. Mach. Learn..

[105] Spataro N, Rodrguez J, Navarro A, Bosch E (2017). Properties of human disease genes and the role of genes linked to Mendelian disorders in complex disease aetiology. Hum. Mol. Genet..

[106] Kipf, T. & Welling, M. Semi-supervised classification with graph convolutional networks. *Proc*. *Int*. *Conf*. *Learn*. *Rep* **5,** 1–14 (2017).

[107] Ying, R. *et al*. Graph Convolutional Neural Networks for Web-Scale Recommender Systems. *Proc*. *ACM SIGKDD Int*. *Conf*. *KDD* **24**, 974–983 (2018).

[108] Wilcoxon F (1945). Individual comparisons by ranking methods. Biometrics Bull..

[109] Jansen, I. *et al*. Genome-wide meta-analysis identifies new loci and functional pathways influencing Alzheimer’s disease risk. *Nat*. *Genet*. **51**, 404–413 (2019). 10.1038/s41588-018-0311-9 PMC6836675 30617256

[110] Kunkle, B. *et al*. Genetic meta-analysis of diagnosed Alzheimer’s disease identifies new risk loci and implicates A*β*, tau, immunity and lipid processing. *Nat*. *Genet*. **51,** 414–430 (2019). 10.1038/s41588-019-0358-2 PMC6463297 30820047

[111] Marioni, R. *et al*. GWAS on family history of Alzheimer’s disease. *Transl*. *Psychiatry* **8,** 99 (2018). 10.1038/s41398-018-0150-6 PMC5959890 29777097

[112] Liu J, Erlich Y, Pickrell J (2017). Case–control association mapping by proxy using family history of disease. Nat. Genet..

[113] Nagele E, Han M, Demarshall C, Belinka B, Nagele R (2011). Diagnosis of Alzheimer’s disease based on disease-specific autoantibody profiles in human sera. Plos One.

[114] Rappaport, N. *et al*. MalaCards: an integrated compendium for diseases and their annotation. *Database* **2013**, 1–14 (2013). 10.1093/database/bat018 PMC3625956 23584832

[115] Raj T (2018). Integrative transcriptome analyses of the aging brain implicate altered splicing in Alzheimer’s disease susceptibility. Nat. Genet..

[116] Sügis, E. HENA project repository. *GitHub*, https://github.com/esugis/HENA (2019).

[117] Hayashi S, Avesani P, Pestilli F (2019). Zenodo.

